# Dermal nonneural granular cell tumor: a case report

**DOI:** 10.1093/jscr/rjac317

**Published:** 2022-07-04

**Authors:** Mohamed Reda El Ochi, Amine Essaoudi, Mohamed Allaoui, Jamal Eddin Abrid, Salima Touri, Nada Moussaoui, Abderrahim El Ktaibi, Amal Damiri, Hafsa Chahdi, Mohamed Oukabli

**Affiliations:** Department of Pathology, Mohammed V Military Hospital, Rabat, Morocco; Faculty of Medicine and Pharmacy of Rabat, Mohammed V University, Rabat, Morocco; Department of Pathology, Mohammed V Military Hospital, Rabat, Morocco; Faculty of Medicine and Pharmacy of Rabat, Mohammed V University, Rabat, Morocco; Department of Pathology, Mohammed V Military Hospital, Rabat, Morocco; Faculty of Medicine and Pharmacy of Rabat, Mohammed V University, Rabat, Morocco; Department of Pathology, Saniat Rmel Hospital, Tetouan, Morocco; Skin Care Center, Tetouan, Morocco; Laboratory of Pathology, Tetouan, Morocco; Department of Pathology, Mohammed V Military Hospital, Rabat, Morocco; Faculty of Medicine and Pharmacy of Rabat, Mohammed V University, Rabat, Morocco; Department of Pathology, Mohammed V Military Hospital, Rabat, Morocco; Faculty of Medicine and Pharmacy of Rabat, Mohammed V University, Rabat, Morocco; Department of Pathology, Mohammed V Military Hospital, Rabat, Morocco; Faculty of Medicine and Pharmacy of Rabat, Mohammed V University, Rabat, Morocco; Department of Pathology, Mohammed V Military Hospital, Rabat, Morocco; Faculty of Medicine and Pharmacy of Rabat, Mohammed V University, Rabat, Morocco

**Keywords:** dermal, nonneural, granular, cell, tumor

## Abstract

Dermal nonneural granular cell tumor is a rare neoplasm of uncertain histogenesis that Le Boit and colleagues originally described in 1991. It arises commonly from the back, extremities and head and neck. To the best of our knowledge, only 50 cases have been reported in adults in the English literature. A 42-year-old man presented with a polypoid skin nodule of the front side of the chest wall, measuring 1,8 × 1,5 cm. The lesion was removed completely with tumor-free margins. Microscopically, the tumor was composed of a diffuse infiltrate of polygonal cells, S 100 negatives, with abundant granular cytoplasm and vesicular nuclei. The diagnosis of dermal nonneural granular cell tumor was retained. No recurrence was noted during follow up of 6 months. The prognosis is good.

## INTRODUCTION

Dermal nonneural granular cell tumors (DNNGCT) are rare neoplasm that Le Boit and colleagues originally described in 1991 [1]. These tumors show no obvious line of differentiation and are composed of large cells with abundant granular cytoplasm [2]. In contrast to conventional granular cell tumor, DNNGCT lacks S100 expression and can exhibit greater nuclear atypia and mitotic activity [ [3]]. We report an additional case in a 42-year-old man.

## CASE REPORT

**Figure 1:**
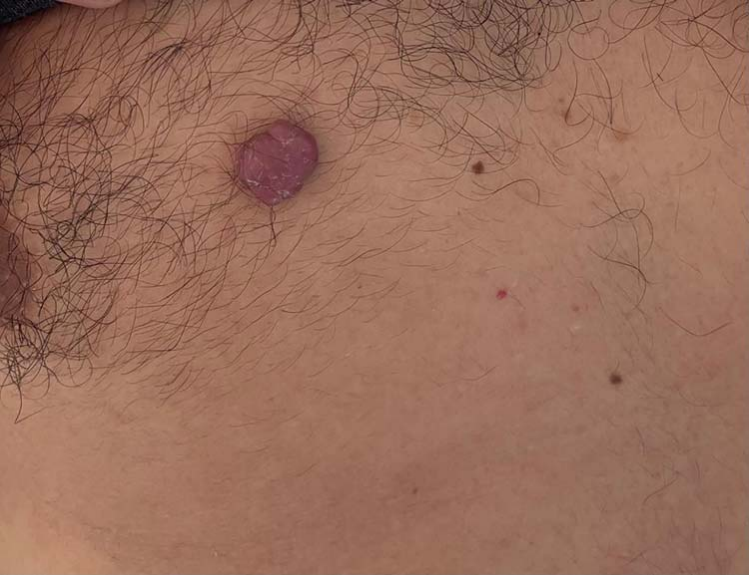
Dermal nonneural granular cell tumor presenting as a non-ulcerated polypoid skin nodule of the front side of the chest wall.

**Figure 2:**
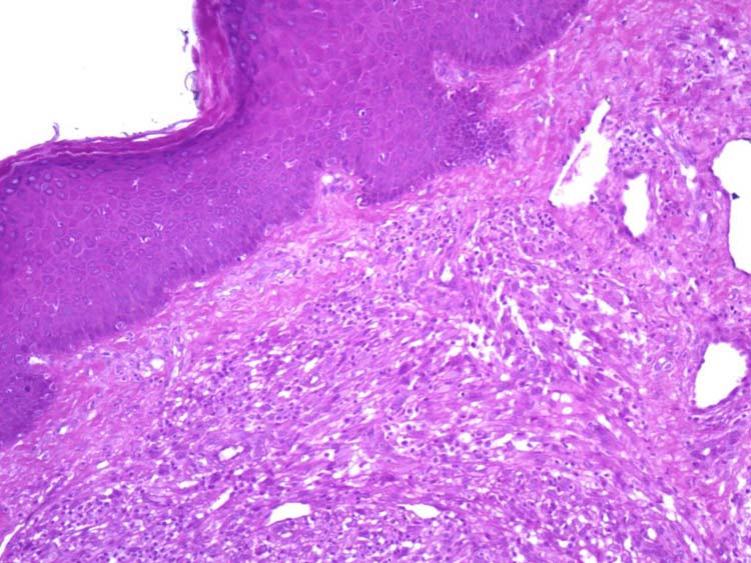
Diffuse infiltrate of polygonal cells (magnification at ×100).

**Figure 3:**
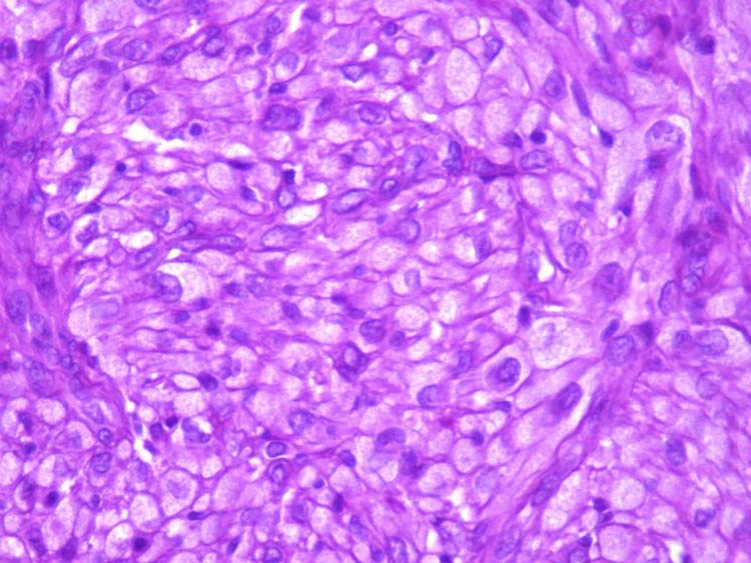
The tumor is composed of epithelioid cells with abundant granular cytoplasm and vesicular nuclei (magnification at ×400).

**Figure 4:**
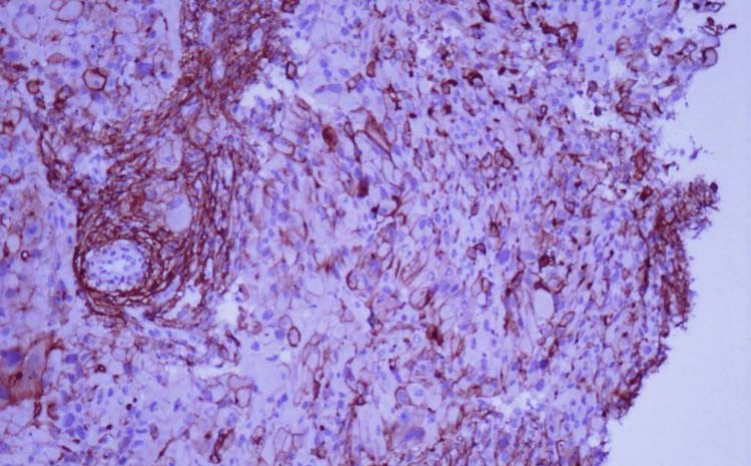
Immunohistochemistry reveals positivity for CD 10 (magnification at ×400).

**Figure 5:**
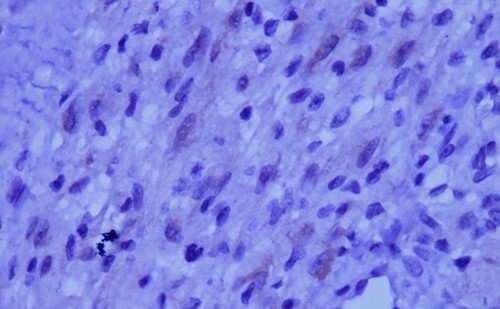
Immunohistochemical staining for ALK is weak and focal (magnification at ×400).

A 42-year-old man with no clinical history presented with a non-ulcerated polypoid skin nodule of the front side of the chest wall, which had grown over a period of 6 months, measuring 1,8 × 1,5 cm (Fig. 1). The lesion was removed completely with tumor-free margins of 0,5 cm. Microscopically, the tumor was composed of a diffuse infiltrate of polygonal cells with abundant granular cytoplasm and vesicular nuclei (Figs 2 and 3). There was no atypia or necrosis. The average mitotic count was one to two per 10 high-power field. Immunohistochemistry showed positive expression for CD10, CD68 and ALK with negative staining for CK AE1/AE3, CD34, S100 and HMB45 (Figs 4 and 5). No recurrence was noted during follow-up of 6 months.

## DISCUSSION

Le boit *et al.* [1] were the first to describe this tumor with negative S100 expression and no clear line of differentiation.

DNNGCT is mainly located on the back, extremities and head and neck [1, 2, 4, 5]. The most common non-cutaneous location is the oral cavity [6]. Studies have shown non-gender preference and an age of distribution ranging from 5 to 83 years, although the majority are young to middle aged (median of 33 years) [1, 2, 4]. No association with clinical or familial condition was reported [7].

The lesion tends to be painless, papulo-nodular or polypoid and may ulcerate. [2]. It is usually small at presentation but can grow to ~1,5 cm [2]. Clinical differential diagnoses include lymphoproliferative processes or melanocytic lesions, such as Spitz nevus or intradermal melanocytic nevus [8].

Microscopically, DNNGCT are well circumscribed and composed of large spindled or polygonal to epithelioid cells with abundant granular cytoplasm [2, 4, 5]. The nuclei are vesicular with a single prominent nucleolus [7]. The mitotic index varies from 1 to 9 per 10 high-power field. A subset of this tumor show atypical cytologic features including nuclear pleomorphism and increased mitoses [1, 2, 4]. These histological features do not seem to correlate with a worse clinical behavior [1, 2, 4].

Immunohistochemistry shows consistent negative expression of S100, smooth muscle actin, desmin, cytokeratin, CD34, HMB 45 and melan-A. CD10 and CD68 are usually positive [2, 3]. ALK antibody can be positive with weak to moderate cytoplasmic expression [6].

ALK gene fusion has been identified in some cases without significant chromosomal gains or losses or additional gene defects [6]. This funding is consistent with the good prognosis of these tumors.

In electron microscopy, the cells have cytoplasmic packed with variably sized secondary lysosomes with no schwanian features [3]. Their precise line of differentiation is unknown [4].

The most important differential diagnoses include conventional granular cell tumors, perivascular epithelioid cell tumors, cellular neurothekeoma and epithelioid cell histiocytoma [6, 7].

Conventional granular cell tumors are poorly circumscribed and have bland nuclei with positivity for S100 [2].

Perivascular epithelioid cell tumors are composed of epithelioid or spindled cells with clear to eosinophilic granular cytoplasm. HMB 45, smooth muscle actin and melan A are generally positive [2].

In cellular neurothekeoma, cytoplasmic granularity and solid growth with little intervening stroma are rare [6].

Epithelioid cell histiocytoma shares several clinicopathological features with DNNGCT. However, prominent granular cytoplasm is generally absent. By immunohistochemistry, both tumors are negative for S100.

The diagnosis of DNNGCT is one of exclusion. Histological evaluation and appropriate immunohistochemical panel are needed before retaining diagnosis.

DNNGCT has an indolent behavior without recurrence or metastasis [3]. Rare cases of regional lymph node metastasis have been reported, but distant spread and lethality have not been seen [3, 4, 9].

In summary, dermal nonneural granular cell tumor is a rare neoplasm with no obvious line of differentiation, composed of large spindled or polygonal to epithelioid cells with abundant granular cytoplasm. In contrast to conventional granular cell tumors, this tumor lacks S100 expression. The diagnosis of this entity is one of exclusion. Histological evaluation and appropriate immunohistochemical panel are needed before retaining diagnosis. The prognosis is good.

## References

[1] Le Boit P , Barr RJ, Burall S, Metcalf JS, Yen TSB, Wick MR. Primitive polypoid granular cell tumour and other cuntaneous granular cell neoplasms of apparent nonneural origin. Am J Surg Pathol 1991;15:48–58. 1985501 10.1097/00000478-199101000-00006

[2] Chaudhry IH , Calonje E. Dermal non-neural cell tumor (so-called primitive polypoid granular cell tumor): a distinctive entity further delineated in a clinicopathological study of 11 cases. Histopathology 2004;47:179–85. 10.1111/j.1365-2559.2005.02192.x 16045779

[3] Habeeb AA , Salama S. Primitive nonneural granular cell tumor (socalled atypical polypoid granular cell tumor). Report of 2 cases with immunohistochemical and ultrastructural correlation. Am J Dermatopathol 2008;30:156–9. 18360120 10.1097/DAD.0b013e318164101c

[4] Lazar AJF , Fletcher CDM. Primitive nonneural granular cell tumors of skin: clinicopatholgic analysis of 13 cases. Am J Surg Pathol 2005;29:927–34. 15958858 10.1097/01.pas.0000157294.55796.d3

[5] Basile JR , Woo SB. Polypoid S-100—negative granular cell tumor of the oral cavity: a case report and review of literature. Oral Surg Oral Med Oral Pathol 2003;96:70–6. 10.1016/s1079-2104(03)00097-0 12847447

[6] Cohen JN , Yeh I, Jordan RC, Wolsky RJ, Horvai AE, McCalmont TH, et al. Cutaneous non-neural granular cell Tumors Harbor recurrent ALK gene fusions. Am J Surg Pathol 2018;42:1133–42. 30001233 10.1097/PAS.0000000000001122

[7] Kabir B , Ramien M, Al Chammary M, de Nanassy J, El Demellawy D. Dermal non-neural granular cell tumor in a 3-year-old child. Pediatr Dermatol 2018;35:241–2. 10.1111/pde.13520 29766548

[8] Torre-Castro J , Moya-Martínez C, Núñez-Hipólito L, Mendoza-Cembranos MD, Eraña-Tomás I, Jo-Velasco M, et al. Three additional cases of non-neural granular cell tumor with novel immunohistochemical findings. J Cutan Pathol 2020;47:1026–32. 32643817 10.1111/cup.13801

[9] Newton P , Schenker M, Wadehra V, Husain A. A case of metastatic nonneural granular cell tumor in a 13-year-old girl. J Cutan Pathol 2014;41:536–8. 24617529 10.1111/cup.12336

